# The limbic-reticular coupling theory of memory processing in the brain and its greater compatibility over other theories

**DOI:** 10.1590/1980-57642018dn12-020002

**Published:** 2018

**Authors:** Zi-Jian Cai

**Affiliations:** 1 CaiFortune Consulting, República Popular da China.

**Keywords:** recall, hippocampus, mammillary body, acetylcholine, gamma wave, recordar, hipocampo, corpo mamilar, acetilcolina, onda gama

## Abstract

The limbic-reticular coupling theory suggests that the hippocampus and amygdala regulate such descending limbic structures as the mammillary bodies, septum, hypothalamus and epithalamus to regulate the ascending noradrenergic, serotonergic, dopaminergic and cholinergic systems, performing declarative memory consolidation and recall. Recent studies have revealed that, less sensitive to familiarity, the hippocampus functions via the fornix, mammillary bodies and hypothalamus for memory recall. Lesions to the thalamic nuclei were complicated with damage to adjacent fornix, stria medullaris and habenula, simultaneously destroying two kinds of structures respectively for familiarity and recall. Furthermore, the orbitofrontal cortex was shown to be clinically irrelevant for memory recall. Electrophysiologically, the hippocampus regulates the raphe nuclei in complex ways, and the hippocampal theta wave activates the dopaminergic cells in ventral tegmental area and cholinergic neurons in basal forebrain, while cholinergic-modulated theta-gamma coupling mediates cortical recall. These concurrent advances support the limbic-reticular coupling theory for elucidation of memory recall.

## Resumo

A teoria do acoplamento límbico-reticular sugere que o hipocampo e a amígdala regulam estruturas límbicas descendentes como os corpos mamilares, *septum*, hipotálamo e epitálamo para regular os sistemas ascendentes noradrenérgico, serotoninérgico, dopaminérgico e colinérgico, realizando a consolidação da memória declarativa e a recordação. Estudos recentes revelaram que, menos sensível à familiaridade, o hipocampo funcionava via fórnice, corpos mamilares e hipotálamo para a recordação da memória. Lesões aos núcleos talâmicos são complicadas com danos ao fórnice, estria medullaris e habenula adjacentes, destruindo simultaneamente dois tipos de estruturas, respectivamente, para familiaridade e recordação. Além disso, o córtex orbitofrontal mostrou-se clinicamente irrelevante para a recordação da memória. Eletrofisiologicamente, o hipocampo regula os núcleos da rafe de maneiras complexas, e a onda teta hipocampal ativa as células dopaminérgicas na área tegmentar ventral e os neurônios colinérgicos no prosencéfalo basal, enquanto que o acoplamento teta-gama colinergicamente modulado medeia a evocação cortical. Esses avanços concorrentes sugerem que a teoria do acoplamento límbico-reticular apropriada para a elucidação da recordação da memória.

Declarative memory is the memory for facts and episodes, and is impaired in amnesia, while procedural memory is the memory for skills and habits, spared in amnesia. Declarative memory can be divided into short-term memory and long-term memory. Short-term memory is the memory of precedent episodes or facts within minutes or seconds, while long-term memory is their recall after hours or days. Further, anterograde memory refers to the long-term memory occurring after the onset of amnesia, while retrograde memory refers to the memory before the onset of amnesia. In addition, recent memory refers to retrograde memory occurring several days to several years before the onset of amnesia, while remote memory refers to memory years before the onset of amnesia. Linguistic words are remote declarative memories.^1^

Bilateral lesions of the medial temporal lobes, including the hippocampus and amygdala, can produce amnesia. In medial temporal lobe amnesia, anterograde memory and recent retrograde memory are impaired, whereas short-term and remote memory are spared. Besides, medial temporal lobe amnesia shows normal release from proactive interference (PI).^2^ There have been many reviews and books discussing the memories impaired and spared in amnesia.^3^^,^ 4

Korsakoff patients are another amnesia group with lesions to the mammillary bodies and the anterior or mediodorsal thalamic nuclei.^5^^,^ 6 The mammillary bodies and anterior thalamic nuclei receive afferents from the hippocampus,^7^^-^ 9 while the mediodorsal thalamic nuclei receive afferents from the amygdala.^9^^,^ 10 In turn, these thalamic nuclei send efferents to the prefrontal and cingulate cortices.^9^^,^ 11 Some diencephalic amnesic patients are additionally impaired in short-term and remote retrograde memory.^12^^,^ 13

There have been many theories attempting to explain the mechanisms of hippocampal memory via its anatomical efferents, which fall into three categories. Notably, one category of theories^3^^,^ 14 suggested the hippocampus and amygdala might mediate memory consolidation and retrieval via their direct efferents to the cortex, with those from the amygdala to the association areas^15^ and those from the hippocampus to the parahippocampal gyrus and entorhinal cortex.^15^ Another notable theory called the disconnection theory^5^^,^ 6 suggests that diencephalic amnesia might result from the disconnection of memory storage sites in the temporal lobe and complex cognition sites in prefrontal cortex following lesions to the thalamus. In the third category, Mishkin and Appenzeller modified the disconnection theory in 1987 by adding the cholinergic (ACh) system in the basal forebrain,^16^ partly accomplishing the biochemical consolidation of memories in cortex with ACh. In parallel, Cai published the limbic-reticular coupling theory in 1990,^4^ suggesting that the hippocampus and amygdala regulate descending limbic structures such as the mammillary bodies, septum, hypothalamus and epithalamus, and in turn regulate the four reticular neuromodulatory systems of noradrenaline (NA), serotonin (5-HT), dopamine (DA) and acetylcholine (ACh), and therefore accomplish declarative memory consolidation and retrieval.^4^ In this article, the author consolidates the latest facts and achievements to compare these theories, and examines their compatibility with recent advances.

## DISSOCIATION OF RECALL AND FAMILIARITY IN VARIOUS AMNESIC PATIENTS

### Lesions to hippocampus and mammillary bodies impair recall but not familiarity

Free recall requires subjects to recollect detailed portions of a memory, while familiarity only requires judgement as to whether a fact or event has previously been encountered without the need to recall the details. Familiarity is the feeling accompanied with recognition manifesting as habituation in sensation and relaxation in response.

Studies on dissociation of free recall and familiarity make it easy to deduce which hippocampal output is responsible for mediation of memory functions. After lesions, the hippocampus and fornix appear vulnerable to free recall and intact in familiarity in humans,^17^^-^ 20 a finding replicated in rats with fornical lesions.^21^

Further downstream in anatomy, through the fornix the hippocampus projects to the mammillary bodies.^7^^,^ 8 Lesions around the mammillary bodies indicate impairments in free recall but not familiarity,^19^^,^ 22 ^,^ 23 resembling the effects of hippocampal/fornical lesions. Lesser damage might not necessarily cause memory impairment.^24^^,^ 25 Moreover, bilateral clinical hypothalamic stimulation elicited autobiographical memories and increased recognition-based recollection rather than familiarity,^26^ resembling hippocampal/fornical memory functions. In contrast, either resection of the anterior temporal lobe^27^ or lesions of the frontal lobe^28^^,^ 29 impaired familiarity but spared recall, the opposite to symptoms in hippocampal/fornical lesions. Accordingly, the hippocampus should accomplish its role in memory recall via its fornical efferents to the mammillary bodies and nearby hypothalamic nuclei, but not via its projections to the temporal or frontal lobe. This conclusion disagrees with a category of theories which hold that the hippocampus mediates its memory functions via its efferents directly to the cortices^3^^,^ 14 (Table 1).

**Table 1:** Comparison of three theories on brain memory processing.

Theories	Anatomy	Supporting evidence	Opposing evidence
Hippocampal efferent memory processing	Hippocampus mediates memory consolidation and recall via its direct efferents to the cortex.	Hippocampus mediates memory consolidation and recall.	(1) Hippocampus mediates free recall, but anterior temporal lobe or frontal lobe does not.(2) Recall in retrosplenial cortex is mediated by theta-gamma (ACh) coupling.
Disconnection theory	The thalamus relays the memories in storage site in temporal lobe and complex cognition site in prefrontal cortex.	Lesions to either medial temporal lobe or thalamus may cause memory impairments.	(1) Hippocampus mediates free recall but not familiarity, whereas the frontal or posterior cingulate cortex does so reversely.(2) Either the anterior or mediodorsal thalamus mediates both familiarity and recall, different from the hippocampus.(3) Recall in retrosplenial cortex is mediated by theta-gamma (ACh) coupling.
Limbic-reticular coupling theory	Hippocampus regulates the mammillary bodies, septum, hypothalamus and epithalamus to regulate the ascending NA, 5-HT, DA and ACh systems to consolidate and recall declarative memories.	(1) Hippocampus, fornix, and mammillary bodies mediate free recall.(2) The hippocampal theta wave activates the DA cells in ventral tegmental area and ACh neurons in basal forebrain.(3) Recall in retrosplenial cortex is mediated by theta-gamma (ACh) coupling.	Currently none available.

### The prefrontal functions in recall and familiarity

The prefrontal cortex, especially the orbitofrontal cortex, is required for the disconnection theory,^5^^,^ 6 and its later modification by Mishkin and Appenzeller.^16^ Nonetheless, clinical studies have not supported its mediation of hippocampal memory functions. It was reported that a patient with extensive damage to bilateral orbitofrontal cortex on MRI exhibited neither memory impairment nor confabulation.^30^ An earlier report also indicated that large bilateral orbitofrontal lesions did not cause amnesia.^31^

Lesions more to other frontal regions often manifest failure to release from proactive interference (PI),^31^^-^ 33 which is spared in hippocampal amnesia.^2^ Besides, recent investigations have demonstrated that, in amnesia with lesions to the hippocampus/fornix, free recall was selectively impaired while familiarity was spared.^17^^-^ 20 By contrast, in patients with frontal lesions, familiarity was impaired while recall remained spared,^28^^,^ 29 the exact opposite symptoms. Obviously, it is not appropriate to adopt the frontal cortex in disconnection theory to mediate the hippocampal functions in memory recall (Table 1).

Nonetheless, many animal experiments have indicated that bilateral lesions of orbitofrontal cortex did cause impairments in memory tasks such as delayed non-matching to sample requiring recall of previous performance.^5^^,^ 16 These discrepancies in human clinical studies are seen in the other behavioral effects of orbitofrontal lesions, in which self-actions/movements had to be remembered. In humans, the orbitofrontal cortex was reported to play roles in self-evaluation and self-reference.^34^^,^ 35 Additionally, damage to the right ventral frontal cortex impaired retrograde memories but not anterograde learning and memory.^36^ Therefore, it was probably the impairments in self-evaluation and reference that might have affected the behavioral tasks in animals. With regard to such controversy, because the clinical results in humans were more accurate in memory division, they are more reliable.

### The pathological duality of diencephalic amnesia

The limbic-reticular coupling theory^4^ predicts that, although the anterior and mediodorsal thalamic nuclei connect the temporal lobe with frontal lobe, damage to the adjacent stria medullaris, habenula and rostral fornix impairs limbic-reticular coupling, thereby also contributing to amnesia. It was reported that, in amnesic cases E.A. and H.J.,^37^ as well as J.W. and B.C.,^38^ the thalamic lesions were restricted only to a small region medial to the mediodorsal thalamic nucleus but not the mediodorsal nucleus itself, which might destroy the stria medullaris and habenula.

In regard to free recall and familiarity, it was demonstrated that the mediodorsal thalamic nucleus was necessary for both recollection and familiarity,^39^ while the anteromedial thalamus was also important for both recall and familiarity,^40^ unlike the fornix or mammillary bodies (Table 1). A more recent paper further showed that Patient 1 with damage to the right anterior thalamic nuclei but not mediodorsal nuclei should have preserved familiarity but did not, while Patient 2 with damage to the right mediodorsal thalamic nuclei but not anterior nuclei should have preserved free recall but did not.^41^ Also, it was reported in rats that, by changing the memory tasks from spatial alteration to sequential contingency learning, the animals with lesions to anterior thalamic nuclei restored their impaired learning ability,^42^ irrelevant for general memory recall.

### Amygdalar sensation of novelty and familiarity

Amygdala plays roles in detection of novelty and familiarity. It has been demonstrated that the activity in human amygdala was increased during presentation of novelty.^43^^,^ 44 Further, it was reported that a patient with hyperperfusion in the right amygdala and hippocampus even misidentified all surrounding persons as her family,^45^ obviously impaired for sensation of novelty. It was also shown that the amygdala was one of the substrates related to false memories,^46^ implying its role in familiarity.

### Other cortices for memory organization and familiarity

There has been growing attention to the memory functions of the retrosplenial cortex. It has been reported that focal lesions to the left retrosplenial cortex causes impairment in acquisition of temporal information^47^ and failure in verbal encoding/categorization,^48^ while lesions to the right retrosplenial cortex result in disorientation in directions but not in buildings and landscapes.^49^ Besides, MRI studies have demonstrated that the retrosplenial cortex was involved in identification of scenes within a large extended environment,^50^ especially relevant to familiar scenes and environment.^51^ Consistently, it was reported that the gamma-power, which was modulated by the reticular ACh neurons,^52^^,^ 53 increased within the human retrosplenial cortex during autobiographical retrieval but not self-referential semantic recall.^54^^,^ 55 Obviously, temporal categorization and spatial orientation correspond to the roles of the retrosplenial cortex (Table 1).

There are few studies relating cingulate lesions to memory impairments in humans, but there are some brain imaging studies in the region. As the cingulate cortex associates the memories in the posterior lobe with the feeling of self from the physiological body, it is likely that the cingulate cortex correlates more to self-memory or familiarity than to contextual recall. Indeed, from human brain imaging studies, the posterior cingulate cortex has been implicated in sensation of familiarity^56^^,^ 57 (Table 1), while the involvement of both the anterior cingulate cortex and retrosplenial cortex in autobiographical memory recall has been implicated.^54^^,^ 55 ^,^ 58 In monkeys, it was demonstrated that anterior cingulate lesions caused less or milder impairments in nonmatching-to-sample and object reversal learning than orbital frontal lesions,^59^^,^ 60 unfavorable for the recall function of the cingulate cortex.

## THE LIMBIC-RETICULAR ELECTROPHYSIOLOGICAL COUPLING

### The limbic-reticular coupling: anatomical facts

In the limbic-reticular coupling theory, the four reticular neuromodulatory systems receive afferents from the descending limbic system.^4^ The locus coeruleus (LC) receives fibers from the central nucleus of amygdala, bed nucleus of stria terminalis, lateral and paraventricular hypothalamic nuclei, lateral preoptic area and also from the raphe nuclei.^61^^,^ 62 The dorsal raphe nucleus receives fibers from the bed nucleus of stria terminalis, nucleus of the diagonal tract, lateral hypothalamus, lateral preoptic area, lateral habenula and also from the LC.^63^^,^ 64 The DA neurons in ventral tegmental area receive afferents from the amygdala, diagonal band of Broca, bed nucleus of stria terminalis, lateral hypothalamus, lateral preoptic area, lateral habenula and also from LC and raphe nuclei.^65^^,^ 66 Primate ACh neurons in the nucleus basalis and diagonal band receive afferents from the amygdala, mammillary bodies, septal nuclei, entorhinal cortex, lateral preoptic area, and also from the LC, raphe nuclei and ventral tegmental area, and so on.^67^

In addition, the mammillary bodies innervate the ascending ACh neurons in the nucleus basalis and diagonal band.^67^ The medial mammillary nucleus also innervates the ventral tegmental area where many ascending DA neurons situate,^68^ while the lateral mammillary nucleus innervates the dorsal tegmental nucleus^68^ which contributes a small portion of ascending ACh projections into the cortex and thalamus.^69^^,^ 70

### Limbic-reticular coupling via the hippocampal theta wave

It is necessary to match the electrophysiological changes in hippocampus to its memory processes. It was demonstrated that the human memory encoding requires theta synchronization,^71^^-^ 73 while memory recall was related to activation in hippocampal CA3 neurons with theta modulation.^74^^,^ 75 It was also shown in rats that, in novel environments for encoding, the theta phase of CA1 shifted closer to the CA1 pyramidal-layer theta peak; while in familiar environments, the theta firing phase shifted closer to the theta trough.^76^

The hippocampal theta wave can propagate to the descending limbic structures. It was shown that neurons in posterior hypothalamic nucleus increased discharge during hippocampal tonic theta, whereas neurons in supramammillary and medial mammillary nucleus displayed rhythmic discharge in phase with the ongoing theta.^77^

It is necessary to correlate the ascending reticular systems to the hippocampus with electrophysiology. It was shown that the theta wave was coupled to the posterior parietal gamma amplitude during human memory recall,^55^^,^ 78 ^,^ 79 while cholinergic blockade reduced the theta-gamma coupling.^80^ The theta-gamma coupling in the retrosplenial cortex^55^ indicated that this region mediates hippocampal recall via cholinergic projections rather than thalamus (Table 1). Moreover, it is important to emphasize that, as demonstrated in the previous section, neither orbitofrontal^30^^,^ 31 nor lateral frontal cortex^28^^,^ 29 is relevant to memory recall. Therefore, it is concluded from these concurrent results that it is the cholinergic-modulated hippocampal theta-gamma coupling that participates in cortical recall of memories.

Recently, a paper published in “Science” illustrated that stimulation of hippocampal CA3 neurons with theta frequency excited the DA neurons in the ventral tegmental area.^74^ It was also shown that the ACh neurons in the basal forebrain were regulated by theta waves.^81^ These results demonstrated that the hippocampal theta activity caused activation of DA neurons in the ventral tegmental area and ACh neurons in the basal forebrain.

The 5-HT neurons cooperate with NA neurons to regulate sleep, arousal and vigilance.^82^^,^ 83 It was reported that the hippocampus and median raphe nucleus manifested theta activities from the medial septum.^84^ The desynchronized hippocampal activity^85^ or activation of CA3 neurons in hippocampus^86^ tended to activate the median raphe nucleus, but inhibited reticular formation.^86^ It is necessary to point out that the dorsal raphe nucleus,^87^ but not the median raphe nucleus,^88^ projects to the cortex. Accordingly, the hippocampus regulates the 5-HT neurons in raphe nuclei in complex ways.

### Other hippocampal activities

The hippocampus also manifests other synchronized and desynchronized activities, such as the place cell activities and so on.^89^ These hippocampal activities are sometimes activated by external cues, and are not unretrieved signals waiting for hippocampus to recall. The unretrieved signals for hippocampus to recall are most likely present together with other signals as hippocampal theta activities, as already known to many people in the media.^90^

The anatomical and electrophysiological facts supporting or disagreeing with the three theories are listed in Table 1.

## DEPICTION OF MEMORY CONSOLIDATION AND RECALL

### Depiction of memory consolidation

Novelty detection and memory encoding cause neuronal activation in the amygdala^43^^,^ 44 ^,^ 73 and theta wave in the hippocampal CA1 region.^73^^,^ 76 Downstream to them, the NA neurons in locus coeruleus(LC) and 5-HT neurons in raphe nuclei increase discharge in response to novelty,^82^^,^ 83 manifesting elevation in vigilance. It has been suggested that the vigilant elevation of their discharge can not only inhibit random firing of cortical neurons to increase signal/noise ratio in attention, but also consolidate the memory of important signals with biochemical effects of NA.^4^^,^ 82 ^,^ 91 ^-^ 93 The dual effects have been postulated as important to temporarily maintain the emotional balance within the limbic system and not to prolong stress afterwards during waking.^91^^-^ 93

In the modified theory of Mishkin,^16^ the ACh system helped biochemical consolidation of memories in cortex, while in the limbic-reticular coupling theory proposed by Cai,^4^ the NA, 5-HT, DA and ACh systems were adopted altogether for this purpose.

**Figure 1:**
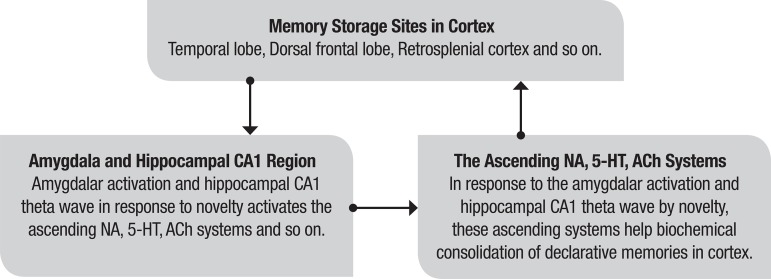
The processes of limbic-reticular coupling theory for memory consolidation.

The processes of limbic-reticular coupling theory for memory consolidation are outlined in Figure 1.

### Depiction of memory recall

Recall of memory activates the hippocampal CA3 neurons with modulation of theta wave,^74^^-^ 76 and in turn activates the ACh neurons in the nucleus basalis/diagonal band and DA neurons in ventral tegmental area through the septum^74^ and mammillary bodies.^67^^,^ 68. In addition, the hippocampal theta-gamma coupling, modulated by the reticular ACh neurons,^52^^,^ 53 participates in human memory recall.^54^^,^ 55 ^,^ 78 ^,^ 79 In parallel, many pharmacological studies have demonstrated that the ACh and DA systems are necessary for free recall in both humans^94^^,^ 95 and animals.^4^^,^ 96

Through activation and modulation of various cortical areas with the ascending ACh and DA systems, via limbic-reticular coupling, a memory cue in hippocampus can synchronously recall several other components of the memory dispersed in other cortical areas. This is the intuitive mechanism by which the hippocampus recalls the memory dispersed in many cortical areas through limbic-reticular coupling.

It could be appropriate to adopt the four ratios ACh/NA, ACh/5-HT, DA/NA and DA/5-HT to depict the brain state favorable for recall or encoding. If the sum of these four ratios ACh/NA, ACh/5-HT, DA/NA, and DA/5-HT increases, the brain state shifts to recall. In contrast, if the sum of the four ratios decreases, the brain state shifts to encoding.

Although very successful for the limbic-reticular coupling theory to explain the hippocampal function of memory, it is still necessary to point out some limitations of this theory: (1) This theory only explains free recall of memory, but not familiarity; (2) This theory only explains the recall of recent memory, but not remote memory; (3) This theory only explains the consolidation of newly acquired declarative memory, but not the mechanism by which recent memory is transformed into remote memory by familiarity, not strictly requiring the hippocampus. These unresolved limitations require the use of the other neural structures in the brain to accomplish them, such as the cingulate cortex, frontal cortex and so forth, which are beyond the scope of this paper.

**Figure 2:**
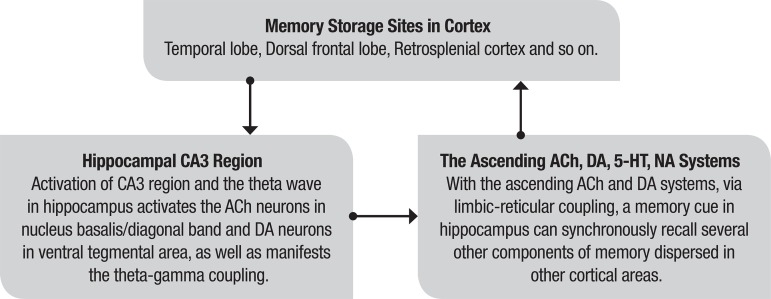
The processes of limbic-reticular coupling theory for memory recall.

The processes of the limbic-reticular coupling theory for recall are outlined in Figure 2.

In conclusion, in this article, we underscore that the hippocampus, fornix and mammillary bodies are involved more in free recall than familiarity, whereby the recall from the hippocampus is relayed to the mammillary bodies via the fornix. However, thalamic lesions are complicated with damage to the nearby fornix, stria medullaris and habenula, being involved in both free recall and familiarity as two distinct pathological mechanisms. Due to its irrelevance to many memory tasks, the orbitofrontal cortex is not an appropriate site for human memory recall, whereas the posterior cingulate cortex may be implicated more in self memory or familiarity.

In the limbic-reticular coupling theory, it was originally suggested in anatomy that the hippocampus and amygdala regulate the descending limbic system and in turn the reticular systems accomplish declarative memory consolidation and recall. Herein, many electrophysiological advances substantiating this theory are cited, especially the relay of hippocampal theta activity containing unretrieved signals for excitation of the ascending DA and ACh neurons; the activation of median raphe nucleus and inhibition of reticular formation by hippocampal CA3 neurons; as well as theta-gamma coupling under reticular ACh modulation participating in human cortical memory recall.

During recall, the hippocampal CA3 neurons are activated with theta wave, and then regulate the ascending ACh and DA systems via limbic-reticular coupling, and in turn via their modulation and effect such as cholinergic-mediated gamma wave, recall many other components of the memory stored in various cortical areas. The biochemical consolidation of memories in cortex is likewise mediated with the ascending ACh, DA, NA and 5-HT systems via limbic-reticular coupling from amygdalar activation and hippocampal CA1 theta wave.
